# Risk Assessment and Virological Monitoring Following an Accidental Exposure to Concentrated Sabin Poliovirus Type 3 in France, November 2018

**DOI:** 10.3390/vaccines8020331

**Published:** 2020-06-22

**Authors:** Marion Jeannoël, Denise Antona, Clément Lazarus, Bruno Lina, Isabelle Schuffenecker

**Affiliations:** 1 Centre National de Référence des Entérovirus et Parechovirus, WHO National Poliovirus Laboratory, Institut des Agents Infectieux, Groupement Hospitalier Nord, Hospices Civils de Lyon, 69317 Lyon, France; marion.jeannoel@chu-lyon.fr (M.J.); bruno.lina@chu-lyon.fr (B.L.); 2 Santé publique France, 94410 Saint Maurice, France; denise.antona@santepubliquefrance.fr; 3 Public Health Emergency Operations Centre, Division of Surveillance and Health Security, General Directorate for Health, Ministry of Solidarity and Health, 75350 Paris, France; Clement.lazarus@sante.gouv.fr; 4 Laboratoire Virpath, Centre International de Recherche en Infectiologie (CIRI), Université de Lyon, Inserm U1111, CNRS UMR 5308, ENS de Lyon, UCBL, 69372 Lyon, France

**Keywords:** poliovirus, incident, accident, risk assessment, containment breach, GAP III

## Abstract

The safe and secure containment of infectious poliovirus (PV) in facilities where live PV are handled is the condition to achieve and maintain poliomyelitis eradication. Despite precautions to minimize the risk of release of PV from such facilities to the environment, breaches of containment have already been documented. Here, we report the management of an incident that occurred on 30 November 2018 in a French vaccine manufacturing plant. Five adequately vaccinated operators were exposed to a Sabin poliovirus type 3 (PV3) spill. A microbiological risk assessment was conducted and the operators were monitored for PV shedding. On day 5 after exposure, Sabin PV3 was detected only in the stool sample of the most exposed worker. Shedding of Sabin PV3 (as detected by viral culture) was restricted to a very short period (less than 15 days). Monitoring of this incident was an opportunity to assess the relevance of our national response plan. We concluded that the measures undertaken and reported here were appropriate and proportional.

## 1. Introduction

Since the World Health Assembly decided to eradicate poliovirus in 1988, wild poliovirus (WPV) cases have decreased by over 99% [1]. Nowadays, 80% of the world’s population is living in certified polio-free regions. In 2019, 176 cases of WPV1, the last WPV still in circulation, were reported (data as of 10th of June 2020), only in parts of Pakistan and Afghanistan and 368 cases of vaccine-derived polioviruses (VDPV) acute flaccid paralysis (AFP) were observed, mostly in Africa (*n* = 321 VDPV2) [2]. Once complete eradication of PV is achieved, infectious PV will remain only in settings such as vaccine manufacturing plants and a few research and diagnostic essential laboratories. The safe and secure containment of infectious PV in such facilities, as required by the Global Action Plan for Poliovirus containment (GAPIII), is the condition to achieve and maintain eradication [3].

Since the beginning of the eradication program, seven documented breaches of containment have been reported, two of them while stringent biorisk management according to GAPIII requirements should be in place (in 2014 and 2017) [4,5,6,7,8].

Here, we report a new incident, with an operator being infected with a Sabin PV3 following a spill in a vaccine manufacturing plant. We describe the management of this incident and discuss the action plan undertaken.

## 2. Materials and Methods

### 2.1. Samples Collection

Pharyngeal and stool specimens were collected from all the exposed workers on day 5 after exposure, and stool specimens were collected on day 15. In case of a documented contamination, it was decided to follow any infected worker every 15 days until the end of PV excretion.

### 2.2. RNA Extraction and rRT-PCR for Detection and Identification of Enterovirus RNA

Viral RNA was extracted using the Nuclisens Easymag platform (Biomérieux, Marcy-l’Etoile, France) following the manufacturer’s protocol. All samples were analyzed by real-time reverse-transcription PCR (rRT-PCR) for generic Enterovirus detection (Enterovirus R-gene^®^, Biomerieux, Marcy-l’Etoile, France). In case of enterovirus detection, VP1 sequencing was performed as described by Nix et al. for serotype identification [9].

### 2.3. Virus Culture for Detection of Infectious Polioviruses

Each sample was inoculated in duplicate on L20B (a mouse cell-line genetically engineered to express the human poliovirus receptor) and rhabdomyosarcoma (RD) cells for poliovirus isolation according to the World Health Organization (WHO) protocol [10]. The L20B cell line was obtained from the National Institute for biological standards and control (NIBSC, Potters bar, United Kingdom) and the RD cell line was obtained from Public Health England (PHE, Porton Down, England). Cytopathic effect (CPE) was monitored daily by microscopy. If no CPE appeared after five days, a blind passage was performed and examination was continued for a further five days. If characteristic CPE appeared, a new passage was performed on the opposite cell-line.

### 2.4. ITD/VDPV RT-PCR for Identification of Poliovirus

The intratypic differentiation (ITD) molecular assay was performed on CPE positive L20B culture supernatants. Viral nucleic acids were extracted from culture supernatants using the Nuclisens Easymag platform and poliovirus detection and typing were performed using the Poliovirus rRT-PCR ITD 5.1 kit and the Poliovirus VDPV 5.0 rRT-PCR kit following the Centers for Disease Control recommendations [11]. 

## 3. Results

### 3.1. Incident

On 30 November 2018, a pipe was accidentally disconnected from a tank containing concentrated Sabin PV3 in a biosafety level 3 (BSL-3) containment area of a vaccine manufacturing plant in France. An initial exposure assessment was performed on site. Five operators were closed to the tank and exposed directly to the spill. All of them wore personal protective equipment (goggles, masks and a one-piece nonwoven overall suit) and undertook a decontamination shower after exposition.

On 3 December, the occupational health practitioner informed local health authorities (Agence Régionale de Santé, ARS) and the National Reference Center for Enteroviruses (NRCEV). The information was immediately relayed to the National Public Health Agency (NPHA) and the Public Health Emergency Operations Center (PHEOC) of the Ministry of Health (MOH). The incident was officially reported by the MOH to the WHO, under the International Health Regulations article 8 (Consultation).

### 3.2. Risk Assessment

A thorough microbiological risk assessment was conducted, involving representatives of the occupational health service of the manufacturer, the NRC EV, the NPHA and the PHEOC. Several meetings were organized and a written microbiological risk assessment was made available on 13 December. 

Regarding the exposed workers, the exposure risk was assessed taking into account that they wore personal protective equipment. The inhalation risk was considered null as all the workers wore masks and their faces were not directly exposed to the spill. The ingestion risk was considered low but not null. All the exposed operators took a decontamination shower as requested in the emergency protocol but the complete effectiveness of the decontamination process could be questioned. As the spill involved a non-neurovirulent vaccine strain and the workers were properly vaccinated and immunized in line with occupational health regulations (complete vaccination scheme with an inactivated poliovirus vaccine (IPV) and serological evidence of protective poliovirus antibody titers confirmed in the last 3 years), the risk of developing poliomyelitis, even if infected, was considered as null. Among household members, no unimmunized or underimmunized persons against polio nor immunosuppressed individuals were identified. More globally, the risk of sustained Sabin PV transmission and VDPV emergence through secondary transmission to the community was considered close to zero. Indeed, the viral load of the earliest positive stool sample was low (see lab results). Additionally, the standard of hygiene is high in France and the sewage system is closed with wastewater treatment at the sewage plant. Lastly, the vaccination coverage is very high (99.1% among children under 2 years and 96% among children in 2018) and well above the level of immunization required to prevent VDPV emergence [12,13]. Also it was estimated that the infected worker did not constitute a higher risk of introduction of an oral polio vaccine (OPV) strain in the community as compared to Sabin PV excreting young children regularly returning in France from geographical areas where National Immunization Days are conducted (e.g., Africa). Yet, through supplementary EV surveillance, no Sabin-like PV has been detected since 2014 and no VDPV has been detected since 2006.

Although the risk of poliomyelitis was considered null for the exposed workers and the risk of transmission to the community very low, it was decided to follow up the viral excretion in the exposed workers. This monitoring was considered to be a real-life test of our national response plan in case of a potential incident involving WPV. The workers were informed they would undergo stool and throat specimens’ collection. Advice on stringent personal hygiene and chlorine disinfection of toilet was given, as well as instructions to avoid contact with persons at high risk of infection (unvaccinated children and immunosuppressed persons). Regarding the infected case (see lab results), it was decided to privatize toilets at work and to maintain chlorine disinfection of toilets and reinforced hand washing until the end of viral excretion. Considering personal needs and the risk of infection for the worker and the community, it was decided neither to isolate the worker at home nor to monitor his close contacts or to test sewage samples. This is in line with the WHO guidance stating that spills involving Sabin PV3 are considered minimal risk situations and that isolation of Sabin PV3 infected persons is not required until OPV cessation [14].

### 3.3. Lab Investigations

**Figure 1:**
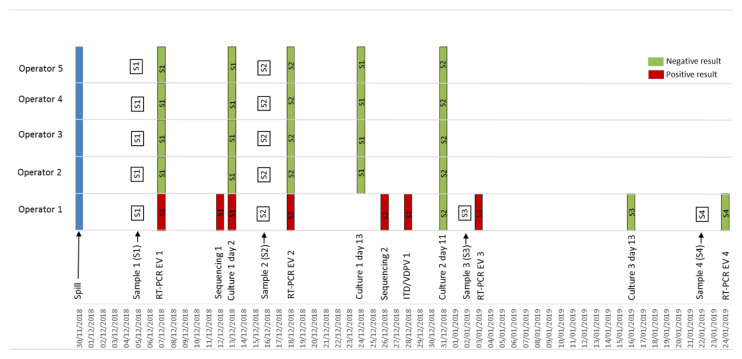
Timeline of the monitoring of vaccine poliovirus-exposed operators following an accidental spill of Sabin PV3, France, November 2018. S1: day 5 samples (throat and stool); S2: day 16 stool samples, S3: day 33 stool sample, S4: day 53 stool sample. The monitoring was performed on the 5 exposed operators from day 5 post-exposure to the end of detection of Sabin PV3 in the stool of the infected operator. Data are presented as a timeline with dates of sampling and tests’ results.

The first batch of samples (day 5 post-exposure) was received at the NRC EV on 5 December 2018. All the specimens were negative by generic Enterovirus rRT-PCR except for the fecal sample of the operator reported to be the most exposed to the spill (Cycle threshold (Ct) = 35). On 12 December, VP1 sequencing of the positive stool sample showed 100% identity to Sabin PV3. On 13 December, virus cultures of the positive sample were positive on both L20B and RD cell-lines. The intratypic differentiation molecular assay performed on the L20B virus culture confirmed the Sabin PV3 identification. Sabin PV3 was detected by rRT-PCR in the infected operator’s stools on day 5 (Ct = 35), 16 (Ct = 36) and 33 (Ct = 40), and detection was finally negative on day 53. L20B/RD cultures were positive only on day 5, in accordance with the low viral load detected by rRT-PCR (Figure 1).

All the results were reported to the Global Poliovirus Laboratory Network (Dr Diop) and to the WHO Regional Office for Europe (Dr Saxentoff).

## 4. Discussion

Here we report on the infection of an operator with a Sabin PV3 strain resulting from a spill in a vaccine manufacturing site in France. This incident underlines the risk of dissemination of PV from facilities handling infectious PV. Despite precautions to minimize the risk of release of PV in the environment, seven documented breaches of containment have already occurred since 1988 (six individuals contaminations and one release of virus in the environment) [4,5,6,7,8]. The latter two occurred while stringent biorisk management rules according to GAPIII requirements should be in place (in 2014 and 2017) [6,7]. They involved neurovirulent strains and areas where some communities have a vaccination coverage <80%, leading to a potential threat of poliomyelitis for underimmunized or immunosuppressed people. In this incident, due to the involvement of a non-neurovirulent strain and to the high vaccination coverage of the French population, the very low risk of poliomyelitis disease allowed to conduct only limited investigations compared to the previous WPV incidents. The only measures taken were the monitoring of the level and duration of viral excretion of the infected operator, combined with the implementation of stringent hygiene measures until the end of the viral excretion. Viral load was low, even in the earliest sample (Day 5) and shedding of infectious virus (cultivable on L20B cells) lasted less than 15 days, compared to the 29-day shedding period observed during the WPV2 incident in the Netherlands [7]. The very limited virus excretion observed in this investigation is probably linked to the lower fitness of the Sabin-like strains compared to wild-type strains. Taking into account all these elements (exposure to a non-neurovirulent strain, low level of shedding, high vaccine coverage, closed sewage system), it was considered that the risk of virus dissemination to household contacts and the community was very low and no additional measures were undertaken.

## 5. Conclusions

Successful polio eradication requires a strict PV containment into PV essential facilities. Accidental spills of PV as described here and previously, are therefore a major concern. It shows that breaches of containment are possible even under GAPIII strict containment rules. Nevertheless, in case of this incident involving a non-neuropathic Sabin strain used in OPV production, excretion duration was demonstrated to be shorter than previously reported in an accident with a WPV strain as used in IPV production [7].

As vaccination coverage level is high in France, this incident was not considered as a public health threat. However, its monitoring was an opportunity to assess the relevance of our national response plan. We concluded that the measures undertaken and reported here were appropriate and proportional.

## References

[1] Poliomyelitis. https://www.who.int/news-room/fact-sheets/detail/poliomyelitis.

[2] GPEI-This Week. http://polioeradication.org/polio-today/polio-now/this-week/.

[3] World Health Organization (2015). WHO Global Action Plan to Minimize Poliovirus Facility-Associated Risk after Type-Specific Eradication of Wild Polioviruses and Sequential Cessation of Oral Polio Vaccine Use: GAPIII.

[4] Mulders M.N., Reimerink J.H., Koopmans M.P., van Loon A.M., van der Avoort H.G. (1997). Genetic Analysis of Wild-Type Poliovirus Importation into The Netherlands (1979–1995). J. Infect. Dis..

[5] Deshpande J.M., Nadkarni S.S., Siddiqui Z.A. (2003). Detection of MEF-1 Laboratory Reference Strain of Poliovirus Type 2 in Children with Poliomyelitis in India in 2002 & 2003. Indian J. Med. Res..

[6] Duizer E., Rutjes S., de Roda Husman A.M., Schijven J. (2016). Risk Assessment, Risk Management and Risk-Based Monitoring Following a Reported Accidental Release of Poliovirus in Belgium, September to November 2014. Euro Surveill..

[7] Duizer E., Ruijs W.L., van der Weijden C.P., Timen A. (2017). Response to a Wild Poliovirus Type 2 (WPV2)-Shedding Event Following Accidental Exposure to WPV2, the Netherlands, April 2017. Euro Surveill..

[8] Bandyopadhyay A.S., Singh H., Fournier-Caruana J., Modlin J.F., Wenger J., Partridge J., Sutter R.W., Zaffran M.J. (2019). Facility-Associated Release of Polioviruses into Communities-Risks for the Posteradication Era. Emerg. Infect. Dis..

[9] Nix W.A., Oberste M.S., Pallansch M.A. (2006). Sensitive, Seminested PCR Amplification of VP1 Sequences for Direct Identification of All Enterovirus Serotypes from Original Clinical Specimens. J. Clin. Microbiol..

[10] World Health Organization (2004). Polio Laboratory Manual.

[11] Kilpatrick D.R., Ching K., Iber J., Chen Q., Yang S.-J., De L., Williams A.J., Mandelbaum M., Sun H., Oberste M.S. (2014). Identification of Vaccine-Derived Polioviruses Using Dual-Stage Real-Time RT-PCR. J. Virol. Methods.

[12] Données de Couverture Vaccinale Diphtérie-tétanos, Poliomyélite, Coqueluche Par Groupe d’âge. https://www.santepubliquefrance.fr/determinants-de-sante/vaccination/donnees-de-couverture-vaccinale-diphterie-tetanos-poliomyelite-coqueluche-par-groupe-d-age.

[13] Wassilak S., Pate M.A., Wannemuehler K., Jenks J., Burns C., Chenoweth P., Abanida E.A., Adu F., Baba M., Gasasira A. (2011). Outbreak of Type 2 Vaccine-Derived Poliovirus in Nigeria: Emergence and Widespread Circulation in an Underimmunized Population. J. Infect. Dis..

[14] World Health Organization (2019). Public Health Management of Facility Related Exposure to Live Polioviruses.

